# Precision (Repeatability and Reproducibility) and Agreement of Corneal Power Measurements Obtained by Topcon KR-1W and iTrace

**DOI:** 10.1371/journal.pone.0147086

**Published:** 2016-01-11

**Authors:** Yanjun Hua, Zequan Xu, Wei Qiu, Qiang Wu

**Affiliations:** Department of Ophthalmology, Shanghai Jiao Tong University Affiliated Sixth People's Hospital, NO.600, Yishan Road, Xuhui District, Shanghai, 200233, China; Bascom Palmer Eye Institute, University of Miami School of Medicine;, UNITED STATES

## Abstract

**Purpose:**

To evaluate the repeatability and reproducibility of corneal power measurements obtained by Topcon KR-1W and iTrace, and assess the agreement with measurements obtained by Allegro Topolyzer and IOLMaster.

**Methods:**

The right eyes of 100 normal subjects were prospectively scanned 3 times using all the 4 devices. Another observer performed additional 3 consecutive scans using the Topcon KR-1W and iTrace in the same session. About one week later, the first observer repeated the measurements using the Topcon KR-1W and iTrace. The steep keratometry (Ks), flat keratometry (Kf), mean keratometry (Km), J0 and J45 were analyzed. Repeatability and reproducibility of measurements were evaluated by the within-subject standard deviation (Sw), coefficient of variation (CoV), test-retest repeatability (2.77Sw), and intraclass correlation coefficient (ICC). Agreements between devices were assessed using Bland-Altman analysis and 95% limits of agreement (LoA).

**Results:**

Intraobserver repeatability and interobserver and intersession reproducibility of the Ks, Kf and Km showed a CoV of no more than 0.5%, a 2.77Sw of 0.70 D or less, and an ICC of no less than 0.99. However, J0 and J45 showed poor intraobserver repeatability and interobserver and intersession reproducibility (all ICCs not greater than 0.446). Statistically significant differences existed between Topcon KR-1W and IOLMaster, Topcon KR-1W and iTrace, Topcon KR-1W and Topolyzer, iTrace and Topolyzer, iTrace and IOLMaster for Ks, Kf and Km measurements (all P < 0.05). The mean differences between Topcon KR-1W, iTrace, and the other 2 devices were small. The 95% LoA were approximately 1.0 D to 1.5 D for all measurements.

**Conclusions:**

The Ks, Kf and Km obtained by Topcon KR-1W and iTrace showed excellent intraobserver repeatability and interobserver and intersession reproducibility in normal eyes. The agreement between Topcon KR-1W and Topolyzer, Topcon KR-1W and IOLMaster, iTrace and Topolyzer, iTrace and IOLMaster, Topcon KR-1W and iTrace were not so good, they should not be interchangeable in clinical application. Given that the intraobserver repeatability and interobserver and intersession reproducibility of corneal astigmatism measurements obtained by Topcon KR-1W and iTrace were poor, it should be cautious that Topcon KR-1W and iTrace were applied for the preparation of toric lens implantation.

## Introduction

Accurate corneal power measurement is essential for managing keratorefractive surgery[1, 2], calculating intraocular lens (IOL) power[3–8], and fitting orthokeratology or customized contact lenses[9–11]. There are several types of devices that can be used for corneal power measurement, such as manual or automated keratometry (e.g., IOLMaster)[2, 12–14], computerized videokeratography based on Placido-disk (e.g., Allegro Topolyzer)[15], Slit-scan system (e.g., Orbscan)[16, 17], Scheimpflug rotating camera system (e.g., Pentacam)[8, 15, 18, 19] and optical coherence tomography (e.g., RTVue100 Fourier-domain OCT)[5, 20, 21]. For all the devices, the corneal power can be calculated by the anterior corneal curvature in certain central corneal surface, the standard corneal refractive index (1.3375) and the refractive index of air (1.000)[22, 23].

The Topcon KR-1W system includes 3 different technologies for analysis of the human eye: wavefront aberrometry based on the Hartmann-Shack principle, Placido-disk corneal topography, and standard auto-refraction[24]. The iTrace system (Tracey Technologies Corp., Houston, TX) uses the principle of ray tracing for obtaining the wavefront aberrations of the eye. In addition to ocular aberrometry, this device has an incorporated Placido-based topographic system that provides corneal topographic maps[25]. Several studies have reported the repeatability and reproducibility of aberrometry obtained by the 2 devices[24–27]. To the best of our knowledge, there have been no reported studies that determined the agreement of corneal power measurements between these 2 devices and other instruments, such as IOLMaster or Allegro Topolyzer, which are widely used in clinical practice.

The purpose of this study was to prospectively assess the intraobserver repeatability and interobserver and intersession reproducibility of corneal power measurements obtained by Topcon KR-1W and iTrace, and then to estimate the agreement of the results obtained by Topcon KR-1W and iTrace with those obtained by IOLMaster and Topolyzer.

## Subjects and Methods

In this prospective study, 100 normal and healthy subjects, including 43 males and 57 females were enrolled. The mean age was 35.11 ± 12.88 years (range 21 to 69 years), and the mean spherical equivalent refraction was -3.00 ± 3.15 diopters (D; range -9.00 to +1.00 D). Only the right eye of each subject was selected for all measurements. This study was conducted in the Department of Ophthalmology, Shanghai Jiaotong University Affiliated Sixth Hospital. All procedures followed the Declaration of Helsinki, and the protocol was approved by the Office of Research Ethical Committee, Shanghai Jiaotong University Affiliated Sixth People’s Hospital. All subjects provided written informed consent after the purpose of the study was explained to them in detail. All subjects could communicate well and cooperated with good fixation ability. Inclusion criteria were healthy subjects with a best corrected distance visual acuity equal to or better than 20/20 and an intraocular pressure of the range of 10 mmHg to 21 mmHg. The exclusion criteria were 1) history of ocular pathology, 2) history of corneal or intraocular trauma, 3) previous ocular surgery, 4) wearing soft contact lenses within 2 weeks or rigid contact lenses within 4 weeks; and 5) dry eye (with subjective dry eye symptoms, tear film break-up time shorter than 5 seconds). Each subject underwent ophthalmic examinations including auto- and manifest-refraction, slit-lamp examination, non-contact intraocular pressure, fundus examination and corneal power measurements with Topcon KR-1W, iTrace, Topolyzer and IOLMaster.

### Instruments and Measurements Protocol

The Topcon KR-1W is an integrated Placido-disk topography and Hartmann-Shack wavefront system that also provides pupillometry, keratometry, and autorefraction in a single measurement[24]. The corneal topographer uses conventional Placido-disk technology to measure corneal curvature with within a range from 5.00 mm to 10.00 mm (in 0.01 mm steps)[25]. It contains 38 Placido rings and measures 13,680 data points, and the keratometry from the central zone of 3 mm diameter were obtained for the analysis. The iTrace uses the principle of ray tracing for wavefront aberration measurements combining Placido-disk based corneal topography[26, 28, 29]. It contains 26 Placido rings and measures 9,360 data points. The Allegro Topolyzer is Placido disk-based videokeratoscope that contains 22 rings and generates high-resolution data of the anterior corneal surface with 22,000 data points. Both iTrace and Allegro Topolyzer obtain keratometric data in three corneal zones: the central zone of 3 mm diameter, the paracentral zone of 5 mm diameter, and the peripheral zone of 7 mm diameter. In the present study, keratometry from the central zone of 3 mm diameter were obtained for the analysis. The IOLMaster is based on the principle of partial coherence interferometry and it measures corneal power by automated keratometry. It uses data from a hexagonal array of 6 points reflected off the anterior corneal surface at a diameter of approximately 2.5 mm, which depends on the corneal curvature.

With all of these devices, the anterior corneal curvature (R_anterior_) in defined central zone is obtained. The standard keratometric index of 1.3375 and the refractive index of air are applied, and corneal power can be calculated using the formula below:

$$Corneal\;power=(1.3375-1.000)/R_{anterior}$$

The repeatability, reproducibility, and agreements were assessed based on those adopted by the British Standards Institute and the International Organization for Standardization[30]. In the first session, observer 1 measured each subject using the 4 devices for the assessment of intraobserver repeatability, and 3 consecutive valid results were obtained for each device. Consequently, Observer 2 obtained 3 additional valid scans using Topcon KR-1W and iTrace for the assessment of interobserver reproducibility. In the second session, one week later Observer 1 obtained another 3 consecutive scans using Topcon KR-1W and iTrace for the assessment of intersession reproducibility, and it was executed at almost the same time as the first session. The sequence of the devices was randomly chosen. All measurements were performed at least 3 hours after waking between 10 am and 5 pm to minimize variations in the results. All the subjects were affirmed to have avoid substantial reading before the measurements[31]. The mean of the 3 scans in the first session obtained by observer 1 for each device was calculated for the assessment of agreement among the 4 devices.

### Statistical Analysis

Statistical analyses were performed using SPSS software for Windows version 17 (SPSS Inc., Chicago, IL, U.S.) and MedCalc Statistical Software version 11.0 (MedCalc Software, Inc., Mariakerke, Belgium). A *p* value of less than 0.05 was considered to have statistical significance. The distribution of all the datasets were analyzed for normality using Kolmogorov-Smirnov tests. For each measurement, the steep keratometry (Ks), the flat keratometry (Kf), the mean keratometry (Km, average of Ks and Kf), and the axis of Ks and Kf were recorded. Corneal astigmatism was converted into a vector Jackson J0 and J45. Calculation were performed with the following formula[32, 33]:

J0=(−cylinder/2)xcos(2xaxis)

J45=(−cylinder/2)xsin(2xaxis)

where the cylinder was the corneal astigmatism magnitude, which was the difference between Ks and Kf, and the axis was the meridian of Ks. These values were calculated for 3 measurements in each session for repeatability, reproducibility and agreement assessment.

To determine the intraobserver repeatability of Topcon KR-1W and iTrace, within-subject standard deviation (Sw), test-retest repeatability (TRT), within-subject coefficient of variation (CoV), and intraclass correlation coefficient (ICC) were calculated for the 3 consecutive measurements obtained during the first session[34]. The test-retest repeatability was defined as 2.77Sw, which indicated the interval within which 95% of the differences between measurements are expected to lie. The CoV was calculated as the ratio of the Sw to the overall mean. A smaller CoV means that the repeatability was higher. It can compare data sets with different units. However, for the data that is near zero, the CoV is too sensitive to have usefulness. In the present study, because both J0 and J45 were close to zero, we didn’t calculate the CoV for them[29, 35]. The ICCs evaluated the consistency for data sets of repeated measurements. If the ICC is closer to 1, the consistency is better. For the assessment of interobserver and intersession reproducibility, CoV, Sw, 2.77Sw and ICCs were also calculated. For the comparison of corneal power measurements obtained by different devices, repeated-measures analysis of variance (ANOVA) with Bonferroni correction was applied to identify pairs that had significant differences. Bland-Altman graphs were plotted to assess the agreement between devices. The 95% limits of agreement (LoA) were defined as ±1.96 standard deviation. A narrower 95% LoA meant better agreement between measurements.

## Results

### Repeatability and Reproducibility of Corneal Power Measurements Obtained by Topcon KR-1W

Table 1 shows the CoV, Sw, 2.77Sw and ICCs for Ks, Kf, Km, J0 and J45 for the three consecutive measurements by 2 observers. The CoV values for Ks, Kf and Km were not greater than 0.34%, and the ICCs of Ks, Kf and Km were greater than 0.99. However, the ICCs of vectors J0 and J45 were not greater than 0.374. Therefore, Topcon KR-1W had high intraobserver repeatability in measuring Ks, Kf and Km, except for J0 and J45.

**Table 1:** The intraobserver repeatability of Ks, Kf, Km, J0 and J45 obtained by Topcon KR-1W. (Note: D: diopter, SD: standard deviation, Sw: within-subject standard deviation, CoV: within-subject coefficient of variation, ICC: intraclass correlation coefficient.)

Parameters	Observers	Mean±SD	CoV (%)	Sw (D)	2.77Sw (D)	ICC
Ks	1st	44.22 ± 1.61	0.23	0.15	0.42	0.997(0.996–0.998)
	2nd	44.22 ± 1.66	0.34	0.24	0.68	0.993(0.990–0.995)
Kf	1st	43.29 ± 1.55	0.19	0.16	0.45	0.996(0.995–0.997)
	2nd	43.31 ± 1.59	0.25	0.14	0.40	0.993(0.990–0.995)
Km	1st	43.78 ± 1.56	0.20	0.14	0.40	0.997(0.996–0.998)
	2nd	43.76 ± 1.60	0.27	0.18	0.50	0.996(0.994–0.997)
J0	1st	-0.002 ± 0.27	-	0.37	1.01	0.374(0.127–0.560)
	2nd	-0.012 ± 0.25	-	0.36	1.00	0.289(0.008–0.500)
J45	1st	-0.031 ± 0.25	-	0.34	0.94	0.363(0.113–0.552)
	2nd	-0.011 ± 0.20	-	0.38	1.06	-0.236(-0.729–0.133)

Table 2 shows the CoV, Sw, 2.77Sw and ICCs for Ks, Kf, Km, J0 and J45 for the assessment of interobserver reproducibility. The CoV values of Ks, Kf, and Km were not more than 0.22%; the ICCs of Ks, Kf and Km were more than 0.99; and the Sw and 2.77Sw values were within 0.20 diopter (D) and 0.57 D. However, the ICCs of J0 and J45 were below 0.45, and the Sw and 2.77Sw values were within 0.22 D and 0.61 D, respectively. The results indicated that Ks, Kf and Km obtained by Topcon KR-1W showed high interobserver reproducibility, except for J0 and J45.

**Table 2:** The interobserver reproducibility of Ks, Kf, Km, J0 and J45 obtained by Topcon KR-1W. (Note: D: diopter, SD: standard deviation, Sw: within-subject standard deviation, CoV: within-subject coefficient of variation, ICC: intraclass correlation coefficient.)

Parameters	Mean±SD	CoV (%)	Sw (D)	2.77Sw (D)	ICC
Ks	44.23 ± 1.63	0.20	0.14	0.40	0.993(0.990–0.995)
Kf	43.30 ± 1.56	0.21	0.20	0.57	0.992(0.987–0.994)
Km	43.77 ± 1.58	0.22	0.16	0.46	0.995(0.992–0.996)
J0	-0.006 ± 0.21	-	0.22	0.61	0.446(0.175–0.628)
J45	-0.021 ± 0.17	-	0.20	0.55	0.352(0.036–0.564)

Table 3 shows the CoV, Sw, 2.77Sw and ICCs for Ks, Kf, Km, J0 and J45 for the assessment of intersession reproducibility. The CoV values of Ks, Kf and Km were not greater than 0.19%; the ICCs were not less than 0.99; and the Sw and 2.77Sw were within 0.09 D and 0.26 D. The ICCs of J0 and J45 were not greater than 0.30, and the Sw and 2.77Sw values were within 0.25 D and 0.70 D, respectively. This indicated that Ks, Kf and Km obtained by Topcon KR-1W had high intersession reproducibility, except for J0 and J45.

**Table 3:** The intersession reproducibility of Ks, Kf, Km, J0 and J45 obtained by Topcon KR-1W. (Note: D: diopter, SD: standard deviation, Sw: within-subject standard deviation, CoV: within-subject coefficient of variation, ICC: intraclass correlation coefficient.)

Parameters	Mean±SD	CoV (%)	Sw (D)	2.77Sw (D)	ICC
Ks	44.19 ± 1.60	0.19	0.09	0.26	0.995(0.993–0.997)
Kf	43.26 ± 1.51	0.16	0.08	0.21	0.990(0.986–0.994)
Km	43.75 ± 1.55	0.16	0.08	0.23	0.995(0.994–0.996)
J0	-0.013 ± 0.16	-	0.25	0.70	0.238(0.024–0.433)
J45	-0.021 ± 0.18	-	0.25	0.70	0.040(-0.485–0.331)

### Repeatability and Reproducibility of Corneal Power Measurements Obtained by iTrace

Table 4 shows the CoV, Sw, 2.77Sw and ICCs for Ks, Kf, Km, J0 and J45 for the three continuous measurements by 2 observers. The CoV values of Ks, Kf and Km were not greater than 0.50%; the ICCs were above 0.99; and the Sw and 2.77Sw were within 0.26 D and 0.70 D. The ICCs of J0 and J45 were not greater than 0.12. The Ks, Kf snd Km obtained by iTrace performed good repeatability, but J0 and J45 didn’t.

**Table 4:** The intraobserver repeatability of Ks, Kf, Km, J0 and J45 obtained by iTrace. (Note: D: diopter, SD: standard deviation, Sw: within-subject standard deviation, CoV: within-subject coefficient of variation, ICC: intraclass correlation coefficient.)

Parameters	Observers	Mean±SD	CoV (%)	Sw (D)	2.77Sw (D)	ICC
Ks	1st	44.03 ± 1.62	0.50	0.26	0.70	0.991(0.988–0.994)
	2nd	44.07 ± 1.63	0.34	0.20	0.55	0.995(0.993–0.996)
Kf	1st	43.02 ± 1.50	0.42	0.21	0.59	0.993(0.991–0.995)
	2nd	43.07 ± 1.51	0.33	0.19	0.53	0.993(0.990–0.995)
Km	1st	43.53 ± 1.53	0.44	0.22	0.61	0.993(0.991–0.995)
	2nd	43.57 ± 1.55	0.32	0.18	0.49	0.996(0.994–0.997)
J0	1st	0.024 ± 0.25	-	0.41	1.14	0.081(-0.283–0.354)
	2nd	-0.019 ± 0.24	-	0.39	1.08	0.111(-0.243–0.376)
J45	1st	-0.006 ± 0.22	-	0.42	1.15	-0.175(-0.629–0.171)
	2nd	0.018 ± 0.27	-	0.40	1.11	-0.257(-0.037–0.478)

Table 5 shows the CoV, Sw, 2.77Sw and ICCs for Ks, Kf, Km, J0 and J45 for the assessment of interobserver reproducibility. The CoV values of Ks, Kf and Km were not greater than 0.30 D, the ICCs were above 0.99, and the Sw and 2.77Sw values were within 0.17 D and 0.47 D, respectively. The ICCs of J0 and J45 were below 0.18; and the Sw and 2.77Sw values were within 0.24 D and 0.66 D. Ks, Kf and Km obtained by iTrace had relatively good reproducibility, but J0 and J45 didn’t.

**Table 5:** The interobserver reproducibility of Ks, Kf, Km, J0 and J45 obtained by iTrace. (Note: D: diopter, SD: standard deviation, Sw: within-subject standard deviation, CoV: within-subject coefficient of variation, ICC: intraclass correlation coefficient.)

Parameters	Mean±SD	CoV (%)	Sw (D)	2.77Sw (D)	ICC
Ks	44.05 ± 1.62	0.30	0.17	0.47	0.994(0.992–0.996)
Kf	42.99 ± 1.54	0.23	0.13	0.35	0.996(0.994–0.998)
Km	43.52 ± 1.53	0.26	0.14	0.38	0.996(0.994–0.997)
J0	0.002 ± 0.18	-	0.23	0.64	0.179(-0.216–0.446)
J45	0.006 ± 0.18	-	0.24	0.66	0.119(-0.312–0.408)

Table 6 shows the CoV, Sw, 2.77Sw and ICCs for Ks, Kf, Km, J0 and J45 for the assessment of intersession reproducibility. The CoV values were no more than 0.36%. The ICCs of Ks, Kf and Km were above 0.99. The Sw and 2.77Sw values of Ks, Kf and Km were within 0.19 D and 0.53 D.

**Table 6:** The intersession reproducibility of Ks, Kf, Km, J0 and J45 obtained by iTrace. (Note: D: diopter, SD: standard deviation, Sw: within-subject standard deviation, CoV: within-subject coefficient of variation, ICC: intraclass correlation coefficient.)

Parameters	Mean±SD	CoV (%)	Sw (D)	2.77Sw (D)	ICC
Ks	43.99 ± 1.62	0.36	0.19	0.53	0.992(0.988–0.995)
Kf	43.01 ± 1.50	0.31	0.18	0.50	0.993(0.989–0.995)
Km	43.50 ± 1.54	0.30	0.17	0.47	0.994(0.990–0.996)
J0	0.016 ± 0.16	-	0.24	0.67	-0.180(-0.762–0.209)
J45	-0.004 ± 0.18	-	0.20	0.57	0.369(0.059–0.576)

### Comparison of Corneal Power Measurements Obtained by Topcon KR-1W, iTrace, Topolyzer and IOLMaster

**Fig 1:**
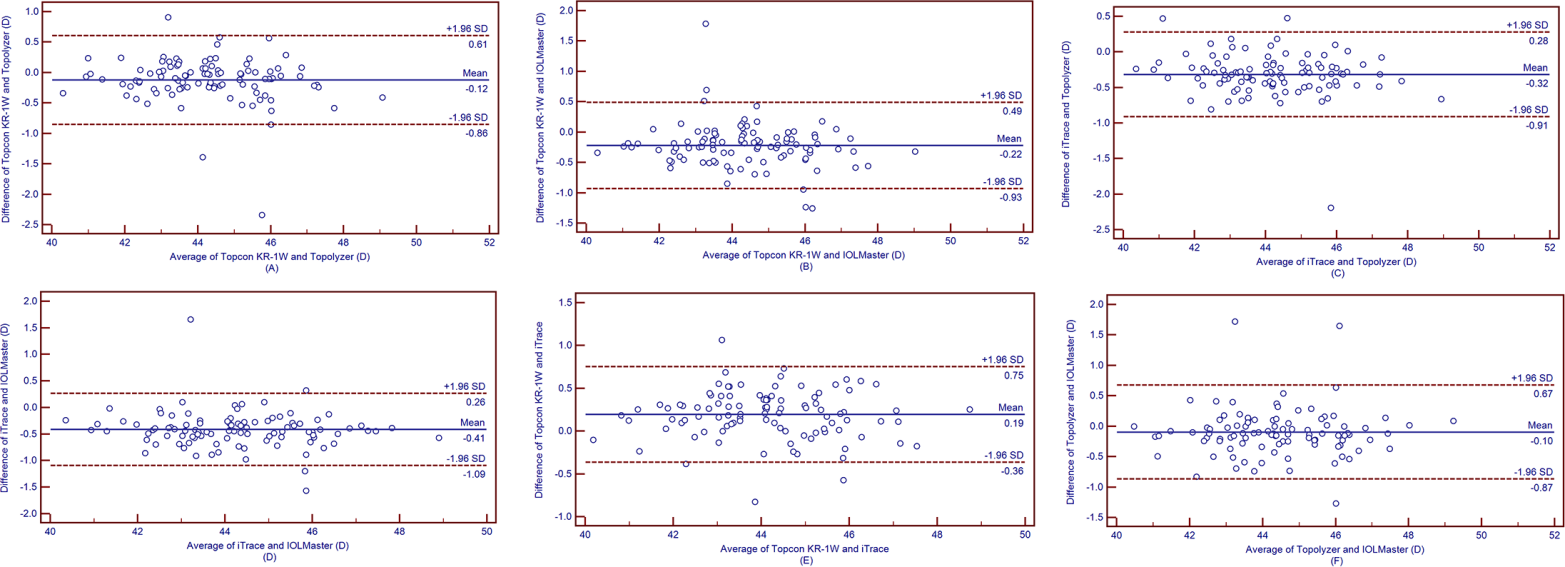
Band-Altman plots present the mean plotted against the differences in values of Ks for a comparison between the Topcon KR-1W and Topolyzer (A), Topcon KR-1W and IOLMaster (B), iTrace and Topolyzer (C), iTrace and IOLMaster (D), Topcon KR-1W and iTrace (E), Topolyzer and IOLMaster (F). The solid line indicates the mean difference. The interval between upper and lower lines represent the 95% LoA.

**Fig 2:**
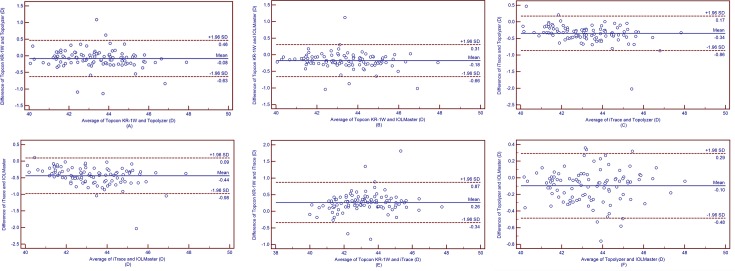
Band-Altman plots present the mean plotted against the differences in values of Kf for a comparison between the Topcon KR-1W and Topolyzer (A), Topcon KR-1W and IOLMaster (B), iTrace and Topolyzer (C), iTrace and IOLMaster (D), Topcon KR-1W and iTrace (E), Topolyzer and IOLMaster (F). The solid line indicates the mean difference. The interval between upper and lower lines represent the 95% LoA.

**Fig 3:**
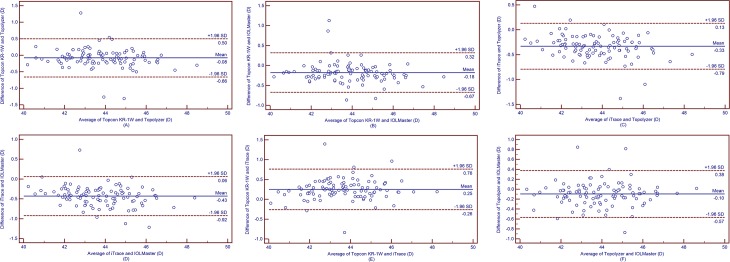
Band-Altman plots present the mean plotted against the differences in values of Km for a comparison between the Topcon KR-1W and Topolyzer (A), Topcon KR-1W and IOLMaster (B), iTrace and Topolyzer (C), iTrace and IOLMaster (D), Topcon KR-1W and iTrace (E), Topolyzer and IOLMaster (F). The solid line indicates the mean difference. The interval between upper and lower lines represent the 95% LoA.

The Ks, Kf and Km values obtained by Topcon KR-1W were significantly smaller than those obtained by IOLMaster (all *p*<0.001, Table 7). The Ks, Kf and Km obtained by Topcon KR-1W were 0.12 D, 0.08 D and 0.08 D smaller than those obtained by Topolyzer (all *p*<0.05, Table 8). The Ks, Kf and Km obtained by iTrace were 0.41 D, 0.44 D and 0.43 D smaller than those obtained by IOLMaster (all *p*<0.001, Table 9). The Ks, Kf and Km obtained by iTrace were 0.32 D, 0.34 D and 0.33 D smaller than those obtained by Topolyzer (all *p*<0.001, Table 10). The Ks, Kf and Km obtained by Topcon KR-1W were 0.19 D, 0.26 D and 0.25 D larger than those obtained by iTrace (all *p*<0.001, Table 11). For Ks measurements, IOLMaster and Topolyzer had comparable results, then Topcon KR-1W followed, and iTrace had the smallest results (*p*< 0.05). For Kf and Km measurements, IOLMaster obtained the largest results, the Topolyzer followed, and then Topcon KR-1W obtained the third, and iTrace obtained the smallest results, respectively (all *p*<0.05). The 95% LoA was relatively wide (close to or more than 1.00 D) in all cases. It means that the agreement among these devices was not good (Figs 1–3).

**Table 7:** Comparison of Ks, Kf and Km obtained by Topcon KR-1W and IOLMaster. (Note: Ks: steep keratometry, Kf: flat keratometry, Km: mean keratometry, D: diopter, SD: standard Error, 95% CI: 95% consistent interval, * Bonferroni corrected.)

Parameters	Mean Difference ± SE	95% CI	*P* value*
Ks(D)	-0.22 ± 0.036	-0.318 to -0.123	< 0.001
Kf(D)	-0.18 ± 0.025	-0.243 to -0.111	< 0.001
Km(D)	-0.18 ± 0.025	-0.245 to -0.109	< 0.001
J0(D)	-	-	-
J45(D)	-	-	-

**Table 8:** Comparison of Ks, Kf and Km obtained by Topcon KR-1W and Topolyzer. (Note: Ks: steep keratometry, Kf: flat keratometry, Km: mean keratometry, D: diopter, SD: standard Error, 95% CI: 95% consistent interval, * Bonferroni corrected.)

Parameters	Mean Difference ± SE	95% CI	*P* value*
Ks(D)	-0.12±0.037	-0.224 to -0.023	< 0.001
Kf(D)	-0.08±0.028	-0.156 to -0.006	0.0268
Km(D)	-0.08±0.029	-0.159 to -0.001	0.0467
J0(D)	-	-	-
J45(D)	-	-	-

**Table 9:** Comparison of Ks, Kf and Km obtained by iTrace and IOLMaster. (Note: Ks: steep keratometry, Kf: flat keratometry, Km: mean keratometry, D: diopter, SD: standard Error, 95% CI: 95% consistent interval, * Bonferroni corrected.)

Parameters	Mean Difference ± SE	95% CI	*P* value*
Ks(D)	-0.41±0.035	-0.507 to -0.321	< 0.001
Kf(D)	-0.44±0.028	-0.514 to -0.367	< 0.001
Km(D)	-0.43±0.025	-0.496 to -0.361	< 0.001
J0(D)	-	-	-
J45(D)	-	-	-

**Table 10:** Comparison of Ks, Kf and Km obtained by iTrace and Topolyzer. (Note: Ks: steep keratometry, Kf: flat keratometry, Km: mean keratometry, D: diopter, SD: standard Error, 95% CI: 95% consistent interval, * Bonferroni corrected.)

Parameters	Mean Difference ± SE	95% CI	*P* value*
Ks(D)	-0.32 ± 0.03	-0.40 to -0.24	< 0.001
Kf(D)	-0.34 ± 0.026	-0.415 to -0.273	< 0.001
Km(D)	-0.33 ± 0.024	-0.395 to -0.268	< 0.001
J0(D)	-	-	-
J45(D)	-	-	-

**Table 11:** Comparison of Ks, Kf and Km obtained by Topcon KR-1W and iTrace. (Note: Ks: steep keratometry, Kf: flat keratometry, Km: mean keratometry, D: diopter, SD: standard Error, 95% CI: 95% consistent interval, * Bonferroni corrected.)

Parameters	Mean Difference ± SE	95% CI	*P* value*
Ks(D)	0.19 ± 0.028	0.117 to 0.270	< 0.001
Kf(D)	0.26 ± 0.031	0.180 to 0.347	< 0.001
Km(D)	0.25 ± 0.026	0.181 to 0.322	< 0.001
J0(D)	-	-	-
J45(D)	-	-	-

## Discussion

There are several Placido-disk based corneal topographers that can be commercially obtained for the application of clinical routines. To the best of our knowledge, no study has comprehensively assessed the intraobserver repeatability and interobserver and intersession reproducibility of corneal power measurements obtained by the 2 Placido-disk based corneal topographers: i.e., Topcon KR-1W and iTrace. In the present study, we evaluated the intraobserver repeatability and interobserver and intersession reproducibility, and agreement in measurements of corneal power and astigmatism by Topcon KR-1W and iTrace, and we then compared the results with those obtained by Topolyzer and IOLMaster. Our data showed good intraobserver repeatability and interobserver and intersession reproducibility of corneal power measurements (i.e., Ks, Kf and Km) obtained by Topcon KR-1W and iTrace, with low Sw (no more than 0.26 D), low CoV (no more than 0.44%), and high ICC values (all above 0.99%). In contrast, the intraobserver repeatability and interobserver and intersession reproducibility of corneal astigmatism measurements (i.e., J0 and J45) obtained by Topcon KR-1W and iTrace were poor with low ICC values (no more than 0.45). This suggested that corneal astigmatism measurements obtained by Topcon KR-1W and iTrace should be viewed with caution in clinical applications because of the poor repeatability and reproducibility of J0 and J45.

In the present study, for the intraobserver repeatability assessment of Ks, Kf and Km, the CoV, 2.77Sw and ICCs of Topcon KR-1W were within 0.19% to 0.34%, 0.40 D to 0.68 D, and 0.993 to 0.997 (Table 1), respectively, compared with those of iTrace which were within 0.32% to 0.50%, 0.49 D to 0.70 D, and 0.991 to 0.996 (Table 4), respectively. For the interobserver reproducibility assessment of Ks, Kf and Km, the CoV values were within 0.20% to 0.22% (Table 2), compared to those within 0.23% to 0.30% (Table 5). For the intersession reproducibility assessment of Ks, Kf and Km, the CoV and 2.77Sw values of Topcon KR-1W were within 0.16% to 0.19%, and 0.21 D to 0.26 D (Table 3), respectively, compared with those of iTrace which were within 0.30% to 0.36%, and 0.47 D to 0.53 D (Table 6), respectively. We concluded that Topcon KR-1W had better repeatability and reproducibility than iTrace for Ks, Kf and Km measurements.

There have been several studies that assessed the repeatability and reproducibility of corneal power measurements obtained by Placido-disc based corneal topographers, and in only a few cases had the repeatability and reproducibility of corneal astigmatism measurements been assessed by means of vector analysis. Mao et al.[36] evaluated the Placido-disk based corneal topographer Keratogragh 4, which had excellent intraobserver repeatability and interobserver and intersession reproducibility. The CoV of all K values were less than 0.3%, and the Sw and 2.77Sw of all parameters were no more than 0.17 D and 0.25 D, respectively, and the ICCs were no more than 0.97. In Huang et al.’s study[35], the intraobserver repeatability and interobserver and intersession reproducibility of all measured parameters showed a CoV of less than 0.24%, a 2.77Sw of 0.29 D or less, and an ICC of more than 0.906. Wang et al.[29] evaluated corneal power measurements from eight devices and found good intraobserver repeatability and interobserver and intersession reproducibility with Medmont E300 (with 32 Placido rings and measuring 9,600 points for every scan), EyeSys Vista (with 26 Placido rings and measuring 9,360 points for every scan) and Allegro Topolyzer. For Medmont E300, the CoV values and 2.77Sw were less than 0.18% and 0.23D, respectively, and ICCs were above 0.997. For EyeSys Vista, the CoV, 2.77Sw and ICCs were less than 0.30%, less than 0.36 D, and above 0.989, respectively. For Allegro Topolyzer, the CoV, 2.77Sw and ICCs were less than 0.29%, less than 0.35 D and above 0.993, respectively. In the present study, the intraobserver repeatability and interobserver and intersession reproducibility of Ks, Kf and Km were relatively comparable to those in the studies mentioned above, but the results of J0 and J45 were not.

In the present study, we compared corneal power measurements obtained by the 4 devices, except for vector J0 and J45 because of their poor repeatability and reproducibility. We found that, for Ks measurements, the results from IOLMaster and Topolyzer were comparable, then those from Topcon KR-1W followed, and those from iTrace was the smallest (Tables 7–11). For Kf and Km measurements, IOLMaster obtained the largest results, then Topolyzer followed, Topcon KR-1W was the third, and then iTrace obtained the smallest results (all *p*<0.05, Tables 7–11). This is in accord with former findings. In previous studies, the repeatability and reproducibility of corneal power measurements obtained by IOLMaster were excellent[29, 37, 38], and it was found that IOLMaster had a little steeper corneal power than Placido disc corneal topography and Scheimpflug camera system[29, 37, 39, 40]. In Wang et al.’s study, the Ks, Kf and Km obtained by IOLMaster were approximately 0.12 D, 0.07 D and 0.10 D higher than those obtained by Topolyzer and Pentacam Scheimpflug camera system, respectively. In our study, the Ks, Kf and Km differences between IOLMaster and Topolyzer were 0.10 D. As is known, the anterior cornea is an aspheric surface in normal eyes, which means that more central corneal zones have steeper corneal power readings[41–43]. IOLMaster takes measurements in a diameter of approximately 2.5 mm of the central cornea, while the other 3 devices measure a diameter of 3 mm. It might be a possible reason why IOLMaster obtained steeper corneal power values than other devices.

We also assessed the agreement of Ks, Kf and Km measurements obtained by the 4 devices using Bland-Altman plots analysis. The 95% LoA of Ks, Kf and Km measurements were within 1.11 D to 1.54 D, 0.77 D to 1.21 D, and 0.92 D to 1.16 D, respectively (Figs 1–3). Huang et al.[35] assessed the agreement of Ks, Kf and Km obtained by a new corneal topographer: i.e., OphthalTop, Pentacam HR Scheimpflug camera and IOLMaster. The 95% LoA of Ks, Kf and Km between OphthalTop and Pentacam HR were 0.72 D, 0.51 D and 0.54 D, and the 95% LoA between OphthalTop and IOLMaster were 0.64 D, 0.55 D and 0.61 D, respectively. Mao et al.[36] evaluated the agreement of corneal power obtained by Keratograph 4, Pentacam HR and IOLMaster. The 95% LoA of Ks, Kf and Km between Keratograph 4 and Pentacam HR were 0.76 D, 0.51 D and 0.56 D, and the 95% LoA between Keratograph 4 and IOLMaster were 0.90 D, 0.42 D and 0.53 D, respectively. As is known, a narrower 95% LoA means better agreement between measurements. For corneal power measurement, a 95% LoA narrower than 1.0 D can be accepted as relatively good agreement, and once the 95% LoA is narrower than 0.5 D, it means that the agreement between measurements is excellent. Therefore, the agreement among Topcon KR-1W, iTrace, Topolyzer and IOLMaster in the present study were not as good as those among OphthalTop and Keratograph 4, Pentacam HR, and IOLMaster in Huang et al ‘s and Mao et al.’s studies[35, 36].

There were several limitations in the present study. First, our study was limited to healthy subjects with normal cornea and good cooperation. The subjects with corneal refractive surgery, keratoconus or other irregular corneas were excluded. Therefore, further studies may require assessment of the performance of Topcon KR-1W and iTrace in corneal power measurements of irregular and postoperative corneas. Second, increasing number of corneal topographers have been applied for corneal power measurements in the clinic: for example, Pentacam Scheimpflug camera system and Fourier-domain OCT. More studies should be carried out to assess the agreement between them.

In conclusion, the Ks, Kf and Km obtained by Topcon KR-1W and iTrace showed excellent intraobserver repeatability and interobserver and intersession reproducibility in normal eyes. The agreement between Topcon KR-1W and Topolyzer, Topcon KR-1W and IOLMaster, iTrace and Topolyzer, iTrace and IOLMaster, Topcon KR-1W and iTrace were not as good, and they should not interchangeable in clinical application. Given that the intraobserver repeatability and interobserver and intersession reproducibility of corneal astigmatism measurements obtained by Topcon KR-1W and iTrace were poor in the present study, cautions should be maintained regarding the applications of Topcon KR-1W and iTrace for the preparation of toric lens implantations.

## Supporting Information

S1 Data Data from Topcon KR-1W. (SAV)

S2 Data Data from iTrace. (SAV)

S3 Data Data from Topolyzer. (SAV)

S4 Data Data from IOLMaster. (SAV)

## References

[1] Liu Y, Wang Y, Wang Z, Zuo T (2012) Effects of error in radius of curvature on the corneal power measurement before and after laser refractive surgery for myopia. Ophthalmic Physiol Opt 32: 355–361. 10.1111/j.1475-1313.2012.00921.x 22697216

[2] Langenbucher A, Scholz K, Szentmary N, Seitz B (2007) Calculations of corneal power after corneo-refractive surgery from keratometry and change of spectacle refraction: some considerations on the "clinical history method". Curr Eye Res 32: 421–429. 17514527 10.1080/02713680701329313

[3] Chan CC, Hodge C, Lawless M (2006) Calculation of intraocular lens power after corneal refractive surgery. Clin Experiment Ophthalmol 34: 640–644. 16970755 10.1111/j.1442-9071.2006.01316.x

[4] De Bernardo M, Capasso L, Rosa N (2014) Algorithm for the estimation of the corneal power in eyes with previous myopic laser refractive surgery. Cornea 33: e2. 10.1097/ICO.0000000000000112 24727635

[5] Huang D, Tang M, Wang L, Zhang X, Armour RL, Gattey DM, et al (2013) Optical coherence tomography-based corneal power measurement and intraocular lens power calculation following laser vision correction (an American Ophthalmological Society thesis). Trans Am Ophthalmol Soc 111: 34–45. 24167323 PMC3797831

[6] Jin H, Auffarth GU, Guo H, Zhao P (2012) Corneal power estimation for intraocular lens power calculation after corneal laser refractive surgery in Chinese eyes. J Cataract Refract Surg 38: 1749–1757. 10.1016/j.jcrs.2012.06.048 22925179

[7] Kalyani SD, Kim A, Ladas JG (2008) Intraocular lens power calculation after corneal refractive surgery. Curr Opin Ophthalmol 19: 357–362. 10.1097/ICU.0b013e3282fec43e 18545021

[8] Kim SW, Kim EK, Cho BJ, Song KY, Kim TI (2009) Use of the pentacam true net corneal power for intraocular lens calculation in eyes after refractive corneal surgery. J Refract Surg 25: 285–289. 19370824 10.3928/1081597X-20090301-08

[9] Joslin CE, Koster J, Tu EY (2005) Contact lens overrefraction variability in corneal power estimation after refractive surgery. J Cataract Refract Surg 31: 2287–2292. 16473219 10.1016/j.jcrs.2005.06.049

[10] Ahmed I, Toufeeq A (2005) Accuracy of intraoperative retinoscopy in corneal power and axial length estimation using a high plus soft contact lens. Ophthalmic Physiol Opt 25: 52–56. 15649183 10.1111/j.1475-1313.2004.00251.x

[11] Haigis W (2003) Corneal power after refractive surgery for myopia: contact lens method. J Cataract Refract Surg 29: 1397–1411. 12900252 10.1016/s0886-3350(02)02044-8

[12] Savini G, Calossi A, Camellin M, Carones F, Fantozzi M, Hoffer KJ (2014) Corneal ray tracing versus simulated keratometry for estimating corneal power changes after excimer laser surgery. J Cataract Refract Surg 40: 1109–1115. 10.1016/j.jcrs.2013.11.032 24874768

[13] Srivannaboon S, Soeharnila, Chirapapaisan C, Chonpimai P (2012) Comparison of corneal astigmatism and axis location in cataract patients measured by total corneal power, automated keratometry, and simulated keratometry. J Cataract Refract Surg 38: 2088–2093. 10.1016/j.jcrs.2012.07.024 22985831

[14] Rosa N, De Bernardo M, Borrelli M, Filosa ML, Minutillo E, Lanza M (2011) Reliability of the IOLMaster in measuring corneal power changes after hyperopic photorefractive keratectomy. J Refract Surg 27: 293–298. 10.3928/1081597X-20100707-01 20672764

[15] Savini G, Barboni P, Carbonelli M, Hoffer KJ (2009) Agreement between Pentacam and videokeratography in corneal power assessment. J Refract Surg 25: 534–538. 19603621 10.3928/1081597X-20090512-07

[16] Arce CG, Campos M, Schor P (2007) Determining corneal power using Orbscan II videokeratography for IOL calculation after excimer laser surgery for myopia. J Cataract Refract Surg 33: 1348–1349; author reply 1349–1350. 17662405 10.1016/j.jcrs.2007.03.062

[17] Leyland M (2004) Validation of Orbscan II posterior corneal curvature measurement for intraocular lens power calculation. Eye (Lond) 18: 357–360. 15069429 10.1038/sj.eye.6700659

[18] Crawford AZ, Patel DV, McGhee CN (2013) Comparison and repeatability of keratometric and corneal power measurements obtained by Orbscan II, Pentacam, and Galilei corneal tomography systems. Am J Ophthalmol 156: 53–60. 10.1016/j.ajo.2013.01.029 23540708

[19] Falavarjani KG, Hashemi M, Joshaghani M, Azadi P, Ghaempanah MJ, Aghai GH(2010) Determining corneal power using Pentacam after myopic photorefractive keratectomy. Clin Experiment Ophthalmol 38: 341–345. 10.1111/j.1442-9071.2010.02286.x 20491804

[20] Tang M, Chen A, Li Y, Huang D (2010) Corneal power measurement with Fourier-domain optical coherence tomography. J Cataract Refract Surg 36: 2115–2122. 10.1016/j.jcrs.2010.07.018 21111315 PMC3005697

[21] Huang D, Swanson EA, Lin CP, Schuman JS, Stinson WG, Chang W, et al (1991) Optical coherence tomography. Science 254: 1178–1181. 1957169 10.1126/science.1957169 PMC4638169

[22] Ho JD, Tsai CY, Tsai RJ, Kuo LL, Tsai IL, Liou SW (2008) Validity of the keratometric index: evaluation by the Pentacam rotating Scheimpflug camera. J Cataract Refract Surg 34: 137–145. 10.1016/j.jcrs.2007.09.033 18165094

[23] Fam HB, Lim KL (2007) Validity of the keratometric index: large population-based study. J Cataract Refract Surg 33: 686–691. 17397744 10.1016/j.jcrs.2006.11.023

[24] Lopez-Miguel A, Martinez-Almeida L, Gonzalez-Garcia MJ, Coco-Martin MB, Sobrado-Calvo P, Maldonado MJ (2013) Precision of higher-order aberration measurements with a new Placido-disk topographer and Hartmann-Shack wavefront sensor. J Cataract Refract Surg 39: 242–249. 10.1016/j.jcrs.2012.08.061 23142546

[25] Pinero DP, Sanchez-Perez PJ, Alio JL (2011) Repeatability of measurements obtained with a ray tracing aberrometer. Optom Vis Sci 88: 1099–1105. 10.1097/OPX.0b013e3182223788 21666525

[26] Pinero DP, Juan JT, Alio JL (2011) Intrasubject repeatability of internal aberrometry obtained with a new integrated aberrometer. J Refract Surg 27: 509–517. 10.3928/1081597X-20101214-01 21188958

[27] Visser N, Berendschot TT, Verbakel F, Tan AN, de Brabander J, Nuijts RM (2011) Evaluation of the comparability and repeatability of four wavefront aberrometers. Invest Ophthalmol Vis Sci 52: 1302–1311. 10.1167/iovs.10-5841 21051697

[28] Molebny VV, Panagopoulou SI, Molebny SV, Wakil YS, Pallikaris IG (2000) Principles of ray tracing aberrometry. J Refract Surg 16: S572–575. 11019876 10.3928/1081-597X-20000901-17

[29] Wang Q, Savini G, Hoffer KJ, Xu Z, Feng Y, Wen D, et al (2012) A comprehensive assessment of the precision and agreement of anterior corneal power measurements obtained using 8 different devices. PLoS One 7: e45607 10.1371/journal.pone.0045607 23049823 PMC3458095

[30] Bland JM, Altman DG (1986) Statistical methods for assessing agreement between two methods of clinical measurement. Lancet 1: 307–310. 2868172

[31] Collins MJ, Buehren T, Bece A, Voetz SC (2006) Corneal optics after reading, microscopy and computer work. Acta Ophthalmol Scand 84: 216–224. 16637840 10.1111/j.1600-0420.2005.00547.x

[32] Thibos LN, Wheeler W, Horner D (1997) Power vectors: an application of Fourier analysis to the description and statistical analysis of refractive error. Optom Vis Sci 74: 367–375. 9255814 10.1097/00006324-199706000-00019

[33] Savini G, Naeser K (2015) An analysis of the factors influencing the residual refractive astigmatism after cataract surgery with toric intraocular lenses. Invest Ophthalmol Vis Sci 56: 827–835. 10.1167/iovs.14-15903 25587061

[34] Bland JM, Altman DG (1996) Measurement error. BMJ 313: 744 8819450 10.1136/bmj.313.7059.744 PMC2352101

[35] Huang J, Savini G, Chen H, Bao F, Li Y, Chen H, et al (2015) Precision and agreement of corneal power measurements obtained using a new corneal topographer OphthaTOP. PLoS One 10: e109414 10.1371/journal.pone.0109414 25559203 PMC4283956

[36] Mao X, Savini G, Zhuo Z, Feng Y, Zhang J, Wang Q, et al (2013) Repeatability, reproducibility, and agreement of corneal power measurements obtained with a new corneal topographer. J Cataract Refract Surg 39: 1561–1569. 10.1016/j.jcrs.2013.04.029 23860010

[37] Shirayama M, Wang L, Weikert MP, Koch DD (2009) Comparison of corneal powers obtained from 4 different devices. Am J Ophthalmol 148: 528–535 e521. 10.1016/j.ajo.2009.04.028 19541287

[38] Vogel A, Dick HB, Krummenauer F (2001) Reproducibility of optical biometry using partial coherence interferometry: intraobserver and interobserver reliability. J Cataract Refract Surg 27: 1961–1968. 11738911 10.1016/s0886-3350(01)01214-7

[39] Savini G, Barboni P, Carbonelli M, Hoffer KJ (2012) Accuracy of corneal power measurements by a new Scheimpflug camera combined with Placido-disk corneal topography for intraocular lens power calculation in unoperated eyes. J Cataract Refract Surg 38: 787–792. 10.1016/j.jcrs.2011.11.037 22386277

[40] Savini G, Barboni P, Carbonelli M, Hoffer KJ (2009) Accuracy of Scheimpflug corneal power measurements for intraocular lens power calculation. J Cataract Refract Surg 35: 1193–1197. 10.1016/j.jcrs.2009.02.031 19545807

[41] Huang J, Pesudovs K, Wen D, Chen S, Wright T, Wang X, et al (2011) Comparison of anterior segment measurements with rotating Scheimpflug photography and partial coherence reflectometry. J Cataract Refract Surg 37: 341–348. 10.1016/j.jcrs.2010.08.044 21241919

[42] Gatinel D, Haouat M, Hoang-Xuan T (2002) [A review of mathematical descriptors of corneal asphericity]. J Fr Ophtalmol 25: 81–90. 11965125

[43] McAlinden C, Khadka J, Pesudovs K (2011) A comprehensive evaluation of the precision (repeatability and reproducibility) of the Oculus Pentacam HR. Invest Ophthalmol Vis Sci 52: 7731–7737. 10.1167/iovs.10-7093 21810981

